# Microbiomes of gall-inducing copepod crustaceans from the corals *Stylophora pistillata* (Scleractinia) and *Gorgonia ventalina* (Alcyonacea)

**DOI:** 10.1038/s41598-018-29953-y

**Published:** 2018-08-01

**Authors:** Pavel V. Shelyakin, Sofya K. Garushyants, Mikhail A. Nikitin, Sofya V. Mudrova, Michael Berumen, Arjen G. C. L. Speksnijder, Bert W. Hoeksema, Diego Fontaneto, Mikhail S. Gelfand, Viatcheslav N. Ivanenko

**Affiliations:** 1 Kharkevich Institute for Information Transmission Problems RAS, B. Karetny per. 19, Moscow, 127051 Russia; 2 Vavilov Institute of General Genetics RAS, Gubkina str. 3, Moscow, 119333 Russia; 3 Center for Data-Intensive Biomedicine and Biotechnology, Skolkovo Institute of Science and Technology, Nobel str. 1, Moscow, 121205 Russia; 4 Faculty of Bioengineering and Bioinformatics, Lomonosov Moscow State University, Moscow, 119992 Russia; 5 Red Sea Research Center, King Abdullah University of Science and Technology (KAUST), Thuwal, 23955 Saudi Arabia; 6 Naturalis Biodiversity Center, Leiden, 2332 AA The Netherlands; 7 National Research Council, Institute of Ecosystem Study, Verbania, 28922 Italy; 8 Faculty of Computer Science, Higher School of Economics, Kochnovsky pr. 3, Moscow, 125319 Russia; 9 Department of Invertebrate Zoology, Biological Faculty, Lomonosov Moscow State University, Moscow, 119992 Russia

## Abstract

Corals harbor complex and diverse microbial communities that strongly impact host fitness and resistance to diseases, but these microbes themselves can be influenced by stresses, like those caused by the presence of macroscopic symbionts. In addition to directly influencing the host, symbionts may transmit pathogenic microbial communities. We analyzed two coral gall-forming copepod systems by using 16S rRNA gene metagenomic sequencing: (1) the sea fan *Gorgonia ventalina* with copepods of the genus *Sphaerippe* from the Caribbean and (2) the scleractinian coral *Stylophora pistillata* with copepods of the genus *Spaniomolgus* from the Saudi Arabian part of the Red Sea. We show that bacterial communities in these two systems were substantially different with *Actinobacteria, Alphaproteobacteria*, and *Betaproteobacteria* more prevalent in samples from *Gorgonia ventalina*, and *Gammaproteobacteria* in *Stylophora pistillata*. In *Stylophora pistillata*, normal coral microbiomes were enriched with the common coral symbiont *Endozoicomonas* and some unclassified bacteria, while copepod and gall-tissue microbiomes were highly enriched with the family ME2 (*Oceanospirillales*) or *Rhodobacteraceae*. In *Gorgonia ventalina*, no bacterial group had significantly different prevalence in the normal coral tissues, copepods, and injured tissues. The total microbiome composition of polyps injured by copepods was different. Contrary to our expectations, the microbial community composition of the injured gall tissues was not directly affected by the microbiome of the gall-forming symbiont copepods.

## Introduction

Coral reefs are one of the most complex marine ecological systems, with biodiversity comparable to that of the rainforests^1,2^. The structural basis of reefs is a complex system, termed coral holobiont, consisting of a core animal, coral polyp, symbiotic unicellular algae of the genus *Symbiodinium*, fungi, protists, viruses and prokaryotes – the coral microbiota^3,4^. With the development of culture-independent methods of high-throughput genome sequencing, the diversity and importance of the coral microbiome for the holobiont fitness has become evident^5–8^, although the direct mechanisms of interactions and functions of the microbial community are not well understood^7,9^. It has been shown that bacteria associated with corals play a role in nitrogen fixation^10^, synthesis of metabolites such as vitamins^11^, cycling of carbon, sulfur, and phosphorus^9^, and resistance to diseases, due to the antibiotic production and to the competition with pathogens for nutrients and space^5,6,9,12^. Moreover, environmental changes have been shown to cause shifts in the coral microbiome composition. Such shifts can be vital for fast adaptation to changing environmental stress conditions^7,12^ and play a role in the evolution of a coral holobiont^7^. On the other hand, changes induced by the stress can shift composition of the coral microbiome toward coral pathogens^13,14^. In particular, considerable changes in the microbiome composition and metabolism accompany bleaching^15–18^, and the microbiome composition is predictive of the corals stress tolerance^19^. Hereby, the establishing of a “healthy” coral microbiome and finding potential etiological agents or groups of agents associated with coral mortality are issues of importance, especially due to the recent degradation of coral reefs induced by human activities and climate shifts^5,20–23^. It is possible, however, that coral mortality is associated not with one group of agents but with the loss of a stable, healthy microbiome and subsequent, diverse stochastic changes in the microbial communities dominated by opportunistic bacteria or r-strategists^24^.

Based on the 16S rRNA gene amplicon massive sequencing, it has been shown that coral prokaryotic communities exhibit almost no overlap with dominating bacterial taxa in the surrounding reef water^3,25–30^ and are usually dominated by *Proteobacteria*, mainly *Gammaproteobacteria* and *Alphaproteobacteria*^31–33^, with different dominating representative genera and species from these classes^32^. Bacteria of the genus *Endozoicomonas* (*Gammaproteobacteria*: *Oceanospirillales*) have been shown to reside in diverse marine hosts varying from sponges to fish all over the world^34^ while being one of the dominant associated taxa of the stony coral *Stylophora pistillata* in the Red Sea^35^ and in temperate gorgonians^36–39^. Other *Oceanospirillales*, such as those of the family ME2, and some *Spirochaetales* have been shown to be the dominant associated taxa in the precious deep-water octocoral *Corallium rubrum* (Linnaeus, 1758)^40^.

Сoral injuries and diseases often lead to shifts in the coral microbial community towards a higher ratio of opportunistic or potentially pathogenic bacteria, like *Rhodobacteraceae*^41–43^ (in particular, *Ruegeria*^44^), Vibrio spp.^42,43,45–48^, *Bacteriodetes*^13^, *Cyanobacteria (Roseofilum reptotaenium*^49,50^ *and Phormidium valderianum*^51^), *Fusobacteria*^13^, *Verrucomicrobiaceae*^47^ and to changes in the species interactions and richness^52,53^. Opportunistic bacteria can come from a variety of sources — they can be minor groups present in the healthy coral, be transmitted with water^54^, or come from adjacent algae microbiomes, being advantageous for algae in the alga-coral competition^55^. One more source of such bacteria can be provided by invertebrates, like copepods. It has been shown that the microbiomes of free-living copepods, which, similar to the microbiomes of corals, are dominated by *Gammaproteobacteria*, *Firmicutes*, *Actinobacteria*, *Cyanobacteria*, and other *Proteobacteria*^56^, may contain bacteria that are potentially pathogenic for corals, such as Vibrio spp.^57,58^. White plague of corals is associated with small crabs of the family Cryptochiridae, which live in small pits or galls inside the host corals^59^, and both the crab and diseased coral microbiomes are dominated by *Alphaproteobacteria*, mainly *Roseobacter*, unlike the microbiomes of healthy corals^60^).

**Figure 1:**
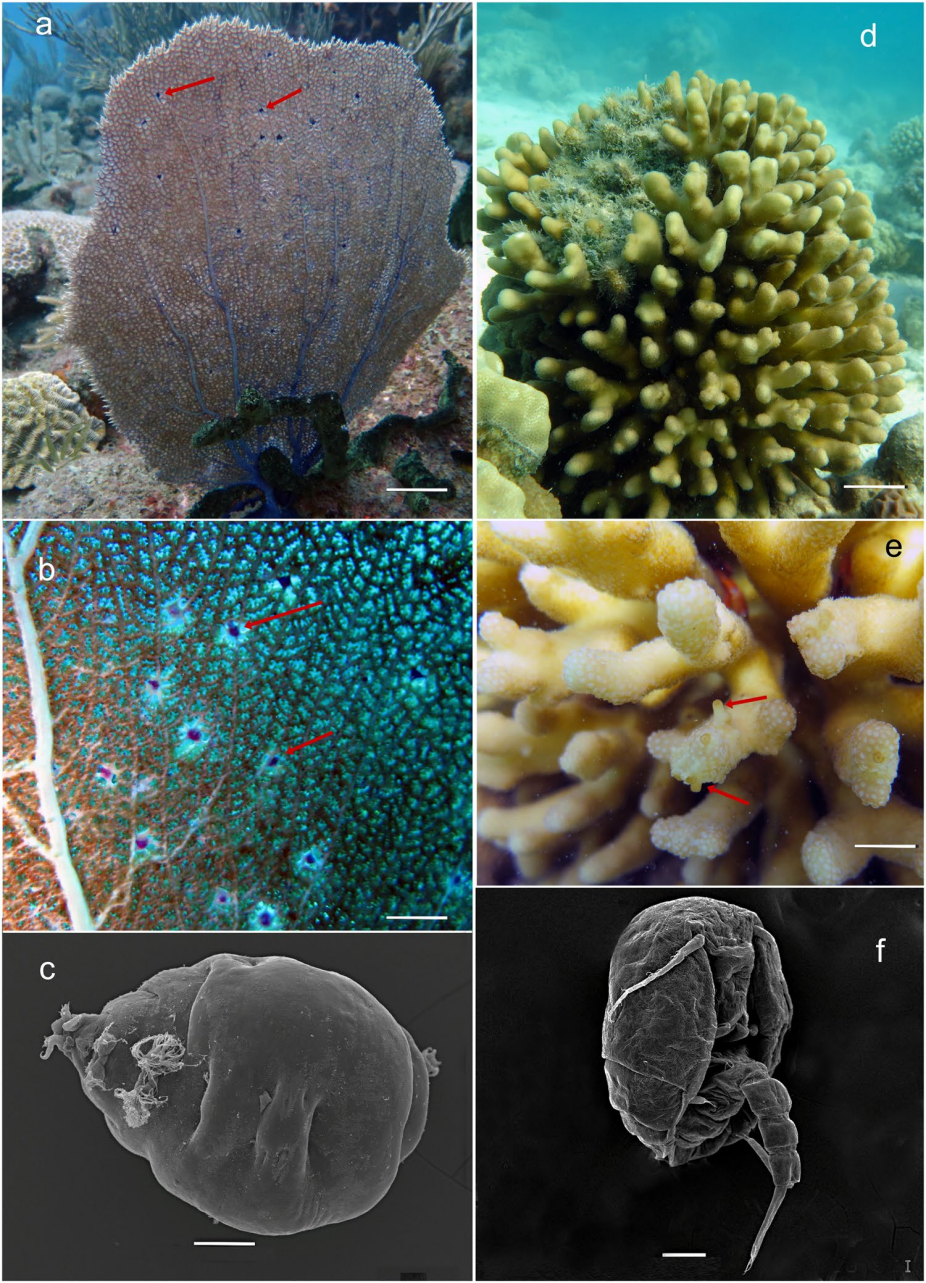
(**a**) The Caribbean sea fan *Gorgonia ventalina* (*Alcyonacea*) with pink galls (**b**, arrowed) induced by a copepod of *Sphaerippe* sp. (*Lamippidae*); (**c**) female of *Sphaerippe* sp., ventral view, SEM photo; (**d**) the Red Sea stony coral *Stylophora pistillata* (*Scleractinia*) with modified corallites (**e**, arrowed) induced by copepods of the genus *Spaniomolgus* (*Rhynchomolgidae*); (**f**) female copepod *Spaniomolgus* sp., ventro-lateral view, SEM photo^65,66^. Scale bars: a–10, b–5, c–0.01, d–8, e–2, f–0.01 cm.

Diverse and abundant symbiotic copepods are found associated with most of the inspected host corals, but the type and strength of the associations are not well-studied^61,62^. Some of the symbiotic copepods have been reported in galls or cysts of corals^63–66^. However, the potential impact of copepods inducing galls to the state of coral hosts remains unknown. Some copepods could potentially act as vectors for the transmission of coral or fish pathogens^67,68^, which in theory might confer benefits for copepods, helping them to overcome host protection mechanisms. In search of such pathogens or microbial complexes specific to symbiotic copepods and coral galls induced by them, we applied 16S rRNA gene metagenomic sequencing to two recently discovered copepod-coral systems (Fig. 1) — the sea fan *Gorgonia ventalina* Linnaeus, 1758 (Anthozoa: Octocorallia: Alcyonacea: Gorgoniidae) with copepods of the genus *Sphaerippe* Grygier, 1980 (Copepoda: Poecilostomatoida: Lamippidae) from the Caribbean island Sint Eustatius^66^ and the scleractinian coral *Stylophora pistillata* Esper, 1797 (Anthozoa: Hexacorallia: Scleractinia: Pocilloporidae) with the copepods of the genus *Spaniomolgus* Humes & Stock, 1972 (Copepoda: Poecilostomatoida: Rhynchomolgidae) from the Saudi Arabian part of the Red Sea^65^. In both systems studied here, copepods were located within a gall or a modified polyp^65,66^. In our knowledge, this is the first detailed analysis of a microbial community of copepods as symbionts of corals. Our expectation is that if copepods are associated with the spread of disease to the coral, the microbiome of the symbiont copepod should share more bacterial species with the diseased coral gall tissue than with the healthy tissues of the same coral colony.

## Results

Overall, about 150,000 reads were obtained per sample (standard deviation 84,500) after chimaera and error checking (Table 1), comprising 54,329 OTUs at the threshold similarity level of 0.987 after removal of singletons and normalization of read numbers by the analysis of rarefaction curves (Figure S1**)**.

**Table 1:** Sample information.

Sample label	Region	Type of sample	RAW reads number	Number of reads after trimming	Number of reads from OTU of size = 1	Number of reads mapped to chloroplast
Gv_g1	Caribbean	*Gorgonia ventalina* gall tissue	279934	254603	9979	227
Gv_g2	Caribbean	*Gorgonia ventalina* gall tissue	156202	142846	7037	87
Gv_h1	Caribbean	*Gorgonia ventalina* normal polyp	178528	162375	6402	25
Gv_h2	Caribbean	*Gorgonia ventalina* normal polyp	199041	183391	6355	1672
Gv_c1	Caribbean	*Sphaerippe* female	130572	118964	6081	364
Gv_c2	Caribbean	*Sphaerippe* female	236283	214948	7651	190
Gv_c3	Caribbean	*Sphaerippe* female	51663	47265	1881	47
Sp_h1	Red Sea	*Stylophora pistillata* normal polyp	119566	108396	4999	6181
Sp_h2	Red Sea	*Stylophora pistillata* normal polyp	41887	38681	1924	875
Sp_h3	Red Sea	*Stylophora pistillata* normal polyp	36701	33686	2307	898
Sp_g1	Red Sea	*Stylophora pistillata* gall tissue	301745	272944	11385	8451
Sp_g2	Red Sea	*Stylophora pistillata* gall tissue	78857	72892	4562	3643
Sp_c1	Red Sea	*Spaniomolgus* female	313308	288680	12756	578
Sp_c2	Red Sea	*Spaniomolgus* female	220848	203011	9364	745
Sp_c3	Red Sea	*Spaniomolgus* female	123227	112850	4764	69

The diversity of the prokaryotic communities expressed as three different metrics of alpha diversity, namely the number of OTUs per sample (ranging from 4,732 to 17,730), the Shannon-Wiener index, and the Simpson index, was not statistically different between the healthy and diseased tissues, and symbiotic copepods for both coral species, nor between the species (Table 2).

**Table 2:** ANOVA analysis of the effect of the coral species (*Gorgonia ventalina* and *Stylophora pistillata*), the type of substrate for the microbiome (healthy tissue, gall tissue, symbiotic copepod), and their interaction, on three different metrics of microbial OTU diversity — species richness, the Shannon index, and the Simpson index. Degrees of freedom (DF), the *F* values, and the *p* values are reported.

Response	Predictor	DF	*F*	*p*
Richness	coral species	1	0.05	0.83
	substrate	2	2.04	0.19
	coral species: substrate	2	2.78	0.12
	Residuals	9		
Shannon	coral species	1	0.44	0.52
	substrate	2	0.09	0.92
	coral species: substrate	2	3.41	0.08
	Residuals	9		
Simpson	coral species	1	0.08	0.79
	substrate	2	0.43	0.66
	coral species: substrate	2	3.95	0.06
	Residuals	9		

The community composition of the samples was significantly different between the coral host species (ADONIS on OTU-based Bray-Curtis distances: *F* = 3.8, *p* = 0.001, *R*^2^ = 0.21), with OTU-based Bray-Curtis distances between the species ranging from 0.64 to 0.96 and within the species from 0.48 to 0.93. No significant differences were observed between the types of substrate for the microbiome (healthy tissues, gall tissues, and symbiotic copepods) (*F* = 1.4, *p* = 0.073, *R*^2^ = 0.15), with distances between the substrates ranging from 0.48 to 0.93, and within from 0.51 to 0.84. The level of similarity between the microbiome types was the same for both coral host species (ADONIS interaction term: *F* = 1.4, *p* = 0.074, *R*^2^ = 0.15).

**Figure 2:**
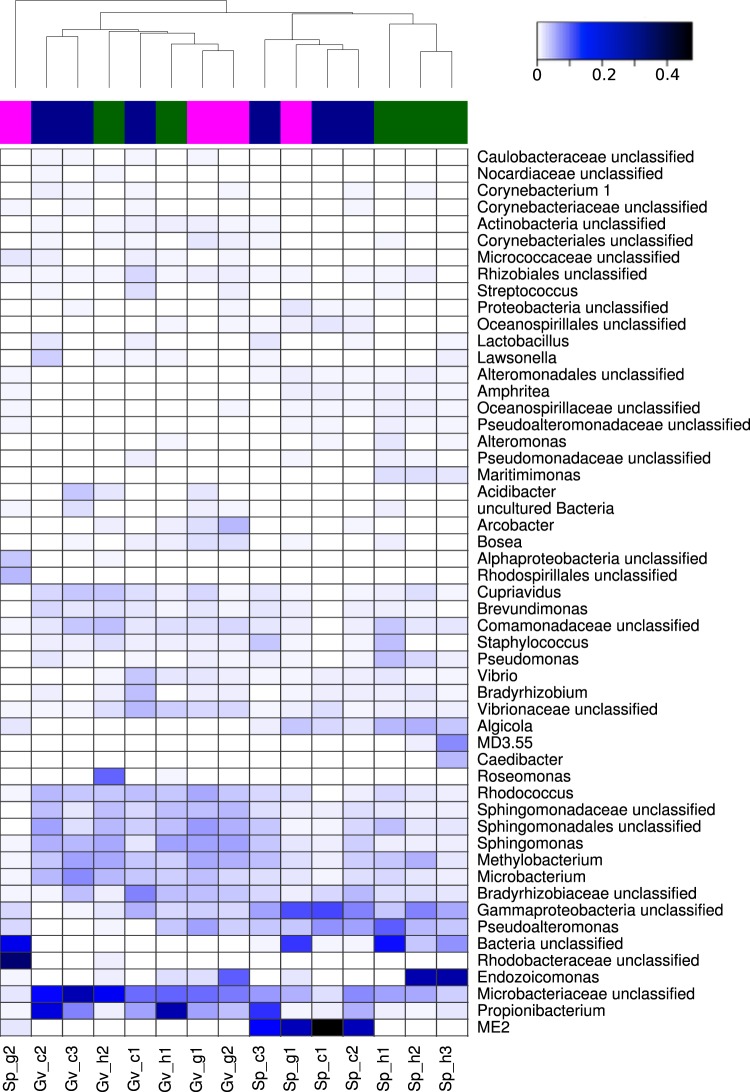
Hierarchical clustering based on relative genus-level taxon abundance. We merge OTUs based on their genus-level taxonomy, if the genus was not identified for an OTU, then the lowest of identified taxonomic category was used. The clustering was based on the Hellinger distances between samples. Only highly abundant taxa are shown. Healthy coral samples are in green, gall samples pink, and copepod samples blue. Abbreviations: Gv_g1, Gv_g2 — gall tissue of the Caribbean sea fan *Gorgonia ventalina* (Alcyonacea); Gv_h1, Gv_h2 — healthy polyp of *G*. *ventalin*; Gv_c1, Gv_c2 and Gv_c3 — female specimens of *Sphaerippe* sp. (Copepoda: Poecilostomatoida: Lamippidae) from galls of *G*. *ventalina*; Sp_h1, Sp_h2 and Sp_h3 — healthy polyp of the Red Sea stony coral *Stylophora pistillata* (Scleractinia); Sp_g1, Sp_g2 — gall tissue (modified polyp) of *S*. *pistillata* from the Red Sea; Sp_c1, Sp_c2 and Sp_c3 — female specimens of *Spaniomolgus* sp. (Copepoda: Poecilostomatoida: Rhynchomolgidae) from gall of *S*. *pistillata*.

Upon considering in detail individual coral colonies, the results were confirmed. The microbial composition of the diseased tissues in *Gorgonia ventalina* was neither significantly different from the healthy tissues in the same coral colony (ADONIS on Bray-Curtis distances: *F* = 0.9, *p* = 0.7, *R*^2^ = 0.31), nor from the microbiome of the symbiotic copepod (*F* = 1.2, *p* = 0.3, *R*^2^ = 0.28). Similarly, the microbial composition of the diseased tissues in *Stylophora pistillata* was neither significantly different from the healthy tissues in the same coral colony (*F* = 1.2, *p* = 0.3, *R*^2^ = 0.29), nor from the microbiome of the symbiotic copepod (*F* = 1.3, *p* = 0.2, *R*^2^ = 0.30). Yet, a plot of the differences between the samples at the genus level suggests some potential effect of the microbiome of the symbiotic copepod on the gall tissues, at least for one gall tissue of *Stylophora pistillata* that clusters with the microbiomes of the symbiotic copepods (Fig. 2). Among three copepod samples of *Gorgonia ventalina*, one clustered with coral samples, both gall and healthy, and two formed a separate cluster. Thus, the microbiomes were highly variable, with an uncertain effect of the symbiotic copepods on the gall tissues.

**Figure 3:**
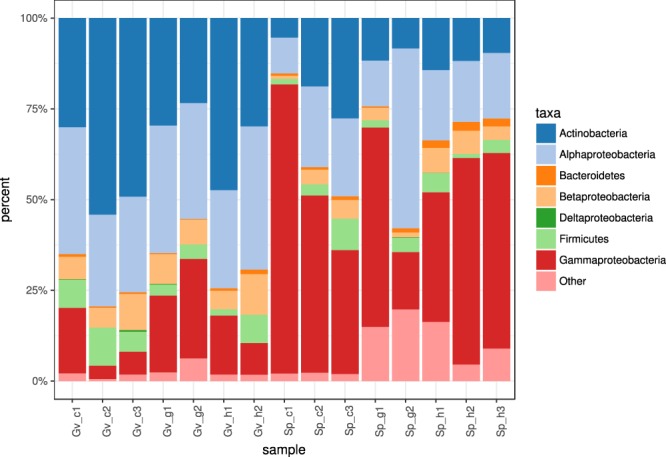
Relative phylum/class abundance in different samples. Abbreviations as in Table 1 and Fig. 2.

**Figure 4:**
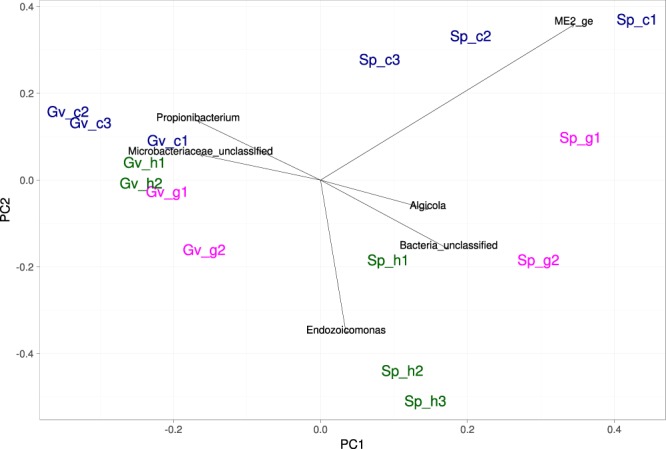
Principal component analysis (PCA) based on the Hellinger distance for all samples. PC1 explains 31% of the variance, and PC2 explains 20% of the variance. Taxa with the largest impact on PC1 and PC2 are shown as arrows. Abbreviations as in Table 1 and Fig. 2.

The main significant difference between the microbiomes was between the coral species systems in two oceans, dominated by different bacterial phyla (Fig. 3). *Actinobacteria*, *Alphaproteobacteria*, and *Betaproteobacteria* were more prevalent in the samples from *Gorgonia ventalina* in the Caribbean, while *Gammaproteobacteria* dominated in the samples from *Stylophora pistillata* in the Red Sea. At the genus level, the PCA visualization confirmed the difference between the coral systems but not by the substrate type (Fig. 4). The main difference between the samples was in the prevalence of *Algicola* and some unclassified bacteria and *Gammaproteobacteria* in all samples from *Stylophora pistillata* with additional very abundant *Oceanospirillales* family ME2 in the gall and copepod samples, while the samples from *Gorgonia ventalina* were rich in *Propionibacterium* and unclassified *Microbacteriaceae*.

The difference in the microbial composition among the copepod samples and the coral samples was mainly generated by *Endozoicomonas*, which was present in all coral samples, and was the dominant taxon in most normal coral samples from the Red Sea, while it was absent or a minority in all copepod samples. Instead, the copepod samples from *Gorgonia ventalina* were enriched in *Propionibacterium* and unclassified *Microbacteriaceae*. On the other hand, the copepod samples from *Stylophora pistillata* together with the gall tissues were enriched in ME2 of *Oceanospirillales*, and the latter was the only taxon that clearly distinguished the copepod and gall samples from the healthy coral samples. No such predominant taxa were observed in the samples from *Gorgonia ventalina*.

## Discussion

Microbiomes of corals and copepods are objects of numerous studies; however, the interactions between copepods and coral microbial communities are poorly understood and have not been studied in detail. The definition of a core healthy coral microbiome meets numerous challenges, the most critical ones being that coral microbial communities are temporally and spatially dynamic^8^, with different coral species possessing different microbiomes^36,40,69^ in different environmental conditions^31,70^. At that, only few studies considered the possibility that copepods can transmit pathogenic bacteria to corals^67^. Such transmitted bacteria can impact the coral health and play a role in the gall formation.

Here we studied bacterial communities of two different copepod–coral associations, focusing on the identification of bacteria possibly involved in the gall formation. These two systems have different microbiomes. The microbiomes of normal corals, galls, and copepods within these systems differ less than between the systems. While we could not distinguish between the influence of geographical location and species-specific host-microbiome interactions due, in particular, to a small number of samples and studied systems, we found that in all metrics, and contrary to initial expectations, the gall samples had a microbiome biodiversity similar to that of the regular coral samples, but with a different microbiome structure. The microbiomes of the normal coral samples from the Red Sea were found to be similar to those previously described in literature^35^ and showed enrichment with widespread coral-associated *Endozoicomonas* bacteria, while the gall samples were enriched with bacteria unusual for regular *Stylophora*, like the family ME2 of *Oceanospirillales*, or by potential pathogens like *Rhodobacteraceae*. The former is normally absent in *Stylophora*, but is one of the dominant taxa in the octocoral *Corallium rubrum*^69^. Similarly, the gall samples from the Caribbean were enriched with potential pathogens like *Arcobacter* and *Pseudoalteromonas*as known to be associated with injured tissues of corals and algae^71,72^. *Oceanospirillales* family ME2 in the Red Sea was not only present in galls but also in all copepods, while absent in the regular coral tissue, which may indicate that these bacteria can be transmitted by copepods and expand in gall tissues. This is reminiscent of the *Roseobacter* prevalence in the microbiomes of the *Cryptochiridae* crabs and white plague coral microbiomes^60^.

Regardless of the large variability in the analyzed microbiomes, no clear evidence of a role of the microbiome associated to the symbiont copepod was found in affecting the microbiome of the gall tissue of the coral. We cannot rule out an influence of the microbiome of the symbiont in the induction of the gall tissue, but its effect is not visible in the microbiome of fully formed coral galls. We acknowledge that our study involved a limited number of samples and of analysed systems, and further studies could still provide evidence of a role of symbiotic copepods in causing or facilitating the spread of disease to corals.

## Materials and Methods

### Field sampling

The corals of *Stylophora pistillata* (Scleractinia) and *Gorgonia ventalina* (Alcyonacea) were collected at the Saudi Arabian Red Sea (25°39′24.49″N, 36°42′43.46″E, Al Wajh Bank, date 01.02.2016, depth 2 m, water T 17 °C) and the Dutch Caribbean island Sint Eustatius (17°27.877′N, 062°58.645′W, date 26.06.2015, depth 6 m, water T 27 °C), respectively^65,66,73,74^.

Each coral was photographed underwater, placed in a separate plastic bag and brought to the surface. The parts of corals with galls were dissected with sterilized needles in sterilized Petri dishes using dissecting microscope Olympus SZX 7, then rinsed several times and preserved in a 95% solution of ethanol. One copepod individual found in the gall was selected per gall of the coral colony. The copepods have been rinsed in ethanol, some of the copepods present in the samples have been inspected by scanning electron microscopy (SEM) in order to detect the presence of microbes.

### Scanning electron microscopy

For scanning electron microscopy (SEM) analyses, copepods were dehydrated through graded ethanol concentrations, critical point dried, mounted on aluminum stubs, coated with gold, and examined in a JEOL scanning electron microscope at the Laboratory of Electron Microscopy (Biological Faculty of Lomonosov Moscow State University)^75^.

### DNA extraction and 16S rRNA gene sequencing

DNA from the ethanol-preserved copepods, galls, or a normal coral tissue was extracted simultaneously using a standard silica-based DNA extraction kit (Diatom DNAprep 100, Isogene, Moscow, Russia). The DNA extraction was conducted according to the manufacturer’s protocol for the fresh blood samples.

Community analysis by 16S-rRNA gene amplicon sequencing targeting the V4 variable region was modified from using universal primers 515F (5′-GTGCCAGCMGCCGCGGTAA-3′) and 806R (5′-GACTACHVGGGTWTCTAAT-3′)^76^. The 515F primer was labeled with sample-specific Multiplex Identification DNA-tags (MIDs) (Table S1).

25 µl PCR reactions contained 12,5 µl 2x Taqman Environmental Mastermix 2.0 (ThermoFisher), 10 pMol/µl V4-F-MID primer and 10 pMol/µl V4-R-trP1 primer (Sigma Aldrich), 1 µl template DNA and PCR grade water. PCR was performed as follows: heated lid 110 °C, 95 °C × 10 mins, followed by 40 cycles of 95 °C × 15 s, 50 °C × 20 s, 60 °C × 30 s, followed by 60 °C × 4 mins and held at 12 °C.

Negative controls with PCR grade water occurred without amplification. PCR products were quantified in the QIAxcel (QIAGEN). PCR products were pooled in an equimolar concentration. The pool was cleaned using AMPure magnetic bead-based purification system (Beckman Coulter). The clean pool was quantified using the Bioanalyser (Agilent). The amplicon library was sequenced using an Ion 314 Chip by an Ion Torrent Personal Genome Machine (Life Technologies) at the Naturalis Biodiversity Center following manufacturer protocol.

### Sequence analysis

The quality of reads was analyzed with FastQC^77^. Long reads were trimmed to 300 bp, first 10 bp and low quality (Phred <20) ends of reads were trimmed, and then reads shorter than 140 bp were removed with Trimmomatic^78^. On average 8.5% of reads were removed. For OTU definition we used CD-HIT-EST^79^ with similarity level 0.987 that was more restrictive than the commonly used 0.97 threshold and was more likely to group together only reads from the same species^80,81^. All single-read OTUs were filtered out (approximately 97500 OTUs accounting for 4.3% of reads). We used two common approaches for accounting for different sequencing depth between samples: (1) normalizing OTU sizes by dividing them by the total number of reads in each sample, and (2) construction of rarefied samples that contained equal numbers of reads by random sub-sampling of the reads. Both methods produced similar results and following^82^ we used normalized OTUs for the beta-diversity analysis. Since some methods for estimating alpha-diversity require absolute numbers, we used rarefaction for all such analyses. Representative sequences from each OTU were scanned for possible chimeras with DECIPHER^83^ and 3056 minor OTUs (that contained less than 2.5% of all reads) were marked as such. We assigned taxonomy to each read the Mothur software package^84^ with standard parameters and SILVA (version 128) as the reference database^85^. If at least 75% of reads from an OTU shared the same taxonomy, it was transferred to the whole OTU. All OTUs classified as chloroplast or eukaryote-related were removed. Rarefaction curves, alpha and beta diversity were calculated using the package vegan for R^86^. To estimate the alpha diversity, we used the Shannon–Wiener index, the Simpson index, and the observed number of species. For the beta diversity analysis, we used the Brain-Curtis dissimilarity and the Hellinger distance, the latter better accounting to low-abundant OTUs. The hierarchical clustering and principal component analyses were performed with built-in R functions based on the OTU or taxon distribution between the samples. Significance of differences between the microbiome compositions was tested with ADONIS implemented in the package vegan for R. The models included the coral holobiont species, the type of substrate for the microbiome (healthy tissue, gall tissue, or symbiont copepod), and the statistical interaction between the coral species and the substrate type.

### Data availability

Sequence data determined in this study are available at NCBI under BioProject Accession PRJNA433804 (http://www.ncbi.nlm.nih.gov/bioproject/433804). Other data are available in the Supplementary Data file.

## Electronic supplementary material

Supplementary information
