# Supplementary material for: Microbiomes of gall-inducing copepod crustaceans from the corals Stylophora pistillata (Scleractinia) and Gorgonia ventalina (Alcyonacea)
Source: Sci Rep. 2018 Aug 1;8:11563. doi: 10.1038/s41598-018-29953-y (PMC6070567; doi:10.1038/s41598-018-29953-y)
Supplement: Supplementary file 1 — Supplementary information [file 41598_2018_29953_MOESM1_ESM.docx]

**Microbiomes of gall-inducing copepod crustaceans from the corals *Stylophora pistillata* (Scleractinia) and *Gorgonia ventalina* (Alcyonacea)**

Pavel V. Shelyakin^1, 2^, Sofya K. Garushyants^1, 3^, Mikhail A. Nikitin^4^, Sofya V. Mudrova^5^, Michael Berumen^5^, Arjen G.C.L. Speksnijder^6^, Bert W. Hoeksema^6^, Diego Fontaneto^7^, Mikhail S. Gelfand^1, 3, 4, 8^, Viatcheslav N. Ivanenko^6, 9, *^

^1^ Kharkevich Institute for Information Transmission Problems RAS, B. Karetny per. 19, Moscow, 127051, Russia

^2^ Vavilov Institute of General Genetics RAS, Gubkina str. 3, Moscow, 119333, Russia

^3^ Center for Data-Intensive Biomedicine and Biotechnology, Skolkovo Institute of Science and Technology, Nobel str. 1, Moscow, 121205, Russia

^4^ Faculty of Bioengineering and Bioinformatics, Lomonosov Moscow State University, Moscow, 119992, Russia

^5^ Red Sea Research Center, King Abdullah University of Science and Technology (KAUST), Thuwal, 23955, Saudi Arabia

^6^ Naturalis Biodiversity Center, Leiden, 2332 AA, The Netherlands

^7^ National Research Council, Institute of Ecosystem Study, Verbania, 28922, Italy

^8^ Faculty of Computer Science, Higher School of Economics, Kochnovsky pr. 3, Moscow, 125319, Russia

^9^ Department of Invertebrate Zoology, Biological Faculty, Lomonosov Moscow State University, Moscow, 119992, Russia

^*^ ivanenko.slava@gmail.com

**Table S1.** Primers used for 16S rRNA sequencing.

| Primer name | Ion Linker | MID | Spacer | 16S Target sequence | Location |  |
| --- | --- | --- | --- | --- | --- | --- |
| V4-F-MID01 | CCATCTCATCCCTGCGTGTCTCCGACTCAG | CTAAGGTAAC | GAT | GTGCCAGCMGCCGCGGTAA | 515f | forward primer |
| V4-F-MID02 | CCATCTCATCCCTGCGTGTCTCCGACTCAG | TAAGGAGAAC | GAT | GTGCCAGCMGCCGCGGTAA | 515f | forward primer |
| V4-F-MID03 | CCATCTCATCCCTGCGTGTCTCCGACTCAG | AAGAGGATTC | GAT | GTGCCAGCMGCCGCGGTAA | 515f | forward primer |
| V4-F-MID04 | CCATCTCATCCCTGCGTGTCTCCGACTCAG | TACCAAGATC | GAT | GTGCCAGCMGCCGCGGTAA | 515f | forward primer |
| V4-F-MID05 | CCATCTCATCCCTGCGTGTCTCCGACTCAG | CAGAAGGAAC | GAT | GTGCCAGCMGCCGCGGTAA | 515f | forward primer |
| V4-F-MID06 | CCATCTCATCCCTGCGTGTCTCCGACTCAG | CTGCAAGTTC | GAT | GTGCCAGCMGCCGCGGTAA | 515f | forward primer |
| V4-F-MID07 | CCATCTCATCCCTGCGTGTCTCCGACTCAG | TTCGTGATTC | GAT | GTGCCAGCMGCCGCGGTAA | 515f | forward primer |
| V4-F-MID08 | CCATCTCATCCCTGCGTGTCTCCGACTCAG | TTCCGATAAC | GAT | GTGCCAGCMGCCGCGGTAA | 515f | forward primer |
| V4-F-MID09 | CCATCTCATCCCTGCGTGTCTCCGACTCAG | TGAGCGGAAC | GAT | GTGCCAGCMGCCGCGGTAA | 515f | forward primer |
| V4-F-MID10 | CCATCTCATCCCTGCGTGTCTCCGACTCAG | CTGACCGAAC | GAT | GTGCCAGCMGCCGCGGTAA | 515f | forward primer |
| V4-F-MID11 | CCATCTCATCCCTGCGTGTCTCCGACTCAG | TCCTCGAATC | GAT | GTGCCAGCMGCCGCGGTAA | 515f | forward primer |
| V4-F-MID12 | CCATCTCATCCCTGCGTGTCTCCGACTCAG | TAGGTGGTTC | GAT | GTGCCAGCMGCCGCGGTAA | 515f | forward primer |
| V4-F-MID13 | CCATCTCATCCCTGCGTGTCTCCGACTCAG | TCTAACGGAC | GAT | GTGCCAGCMGCCGCGGTAA | 515f | forward primer |
| V4-F-MID14 | CCATCTCATCCCTGCGTGTCTCCGACTCAG | TTGGAGTGTC | GAT | GTGCCAGCMGCCGCGGTAA | 515f | forward primer |
| V4-F-MID15 | CCATCTCATCCCTGCGTGTCTCCGACTCAG | TCTAGAGGTC | GAT | GTGCCAGCMGCCGCGGTAA | 515f | forward primer |
| V4-F-MID16 | CCATCTCATCCCTGCGTGTCTCCGACTCAG | TCTGGATGAC | GAT | GTGCCAGCMGCCGCGGTAA | 515f | forward primer |
| V4-F-MID17 | CCATCTCATCCCTGCGTGTCTCCGACTCAG | TCTATTCGTC | GAT | GTGCCAGCMGCCGCGGTAA | 515f | forward primer |
| V4-F-MID18 | CCATCTCATCCCTGCGTGTCTCCGACTCAG | AGGCAATTGC | GAT | GTGCCAGCMGCCGCGGTAA | 515f | forward primer |
| V4-F-MID19 | CCATCTCATCCCTGCGTGTCTCCGACTCAG | TTAGTCGGAC | GAT | GTGCCAGCMGCCGCGGTAA | 515f | forward primer |
| V4-F-MID20 | CCATCTCATCCCTGCGTGTCTCCGACTCAG | CAGATCCATC | GAT | GTGCCAGCMGCCGCGGTAA | 515f | forward primer |
| V4-R-trP1 | CCTCTCTATGGGCAGTCGGT |  | GAT | GGACTACHVGGGTWTCTAAT | 806r | reverse primer |


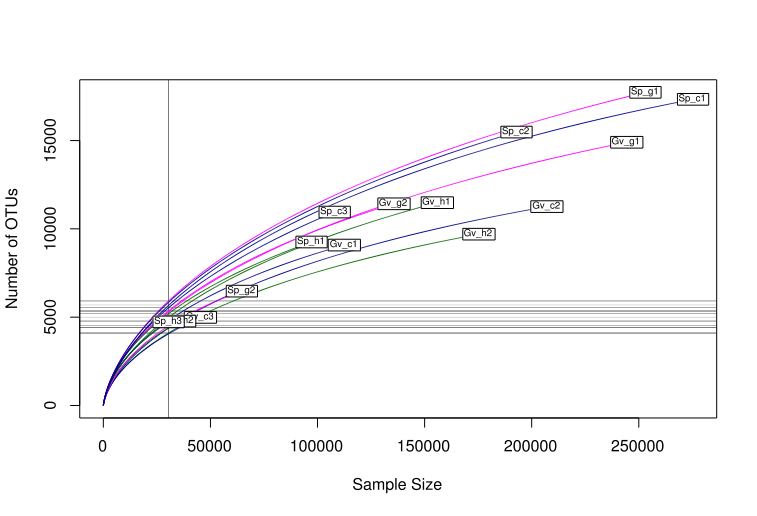


**Figure S1.** Rarefaction curves for all samples. OTUs are clustered at the 98.7% identity level. The vertical line marks the number of reads in the smallest sample, the horizontal lines mark the numbers of OTUs clustered in each sample after sub-sampling of reads to equalize the sample sizes. Healthy coral samples are in green, gall samples pink, and copepod samples **blue.** Abbreviations: Gv_g1, Gv_g2 — gall tissue of the Caribbean sea fan *Gorgonia ventalina* (Alcyonacea); Gv_h1, Gv_h2 — healthy polyp of *G. ventalin*; Gv_c1, Gv_c2 and Gv_c3 — female specimens of *Sphaerippe* sp. (Copepoda: Poecilostomatoida: Lamippidae) from galls of *G. ventalina;* Sp_h1, Sp_h2 and Sp_h3 — healthy polyp of the Red Sea stony coral *Stylophora pistillata* (Scleractinia); Sp_g1, Sp_g2 — gall tissue (modified polyp) of *S. pistillata* from the Red Sea; Sp_c1, Sp_c2 and Sp_c3 — female specimens of *Spaniomolgus* sp. (Copepoda: Poecilostomatoida: Rhynchomolgidae) from gall of *S. pistillata*.


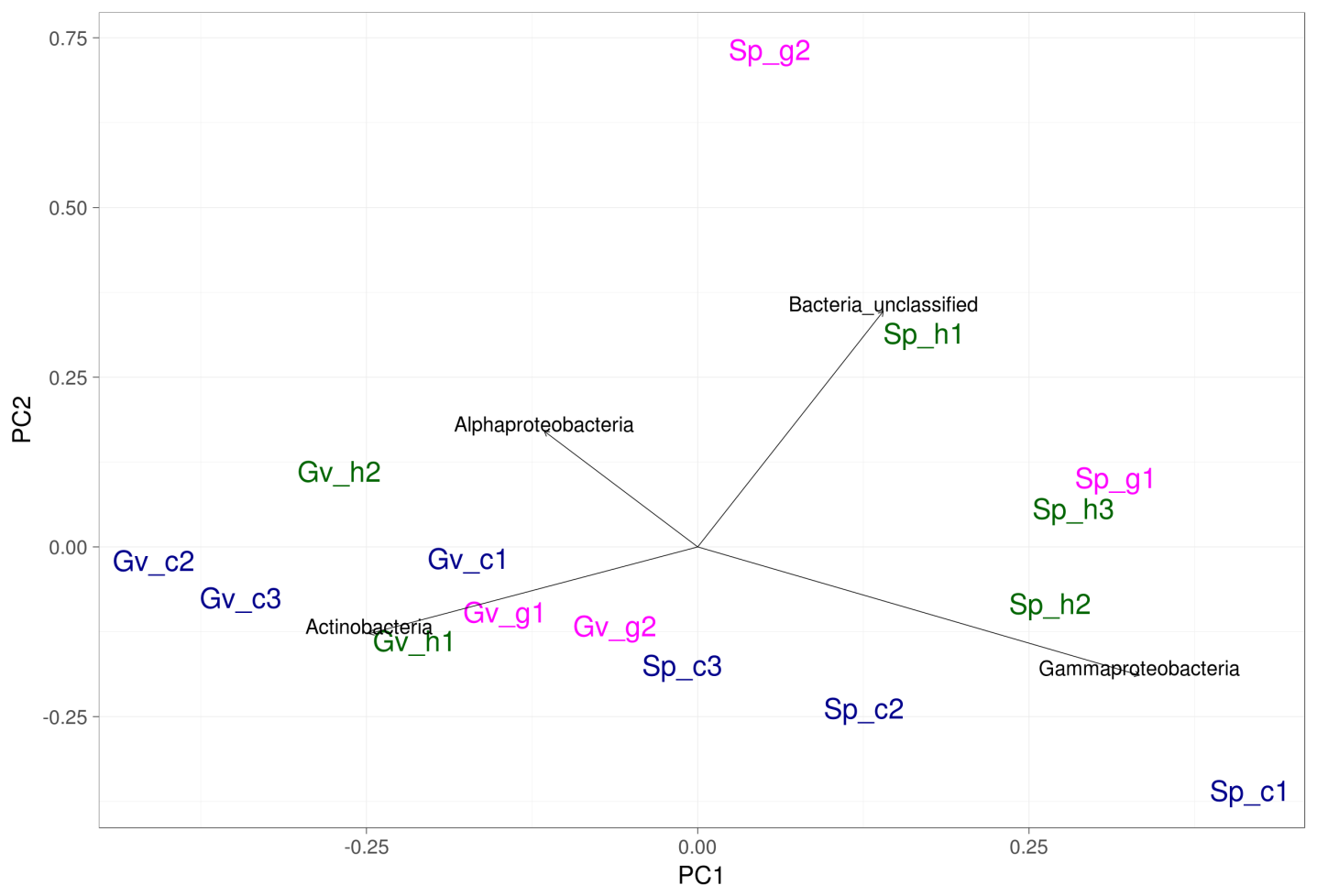
 **Figure S2.** Principal component analysis (PCA) for all samples based on the Hellinger distance and class-level taxonomy. For PCA we merge OTU based on their class-level taxonomy, if the class was not identified for an OTU, then the lowest of identified taxonomic category was used. PC1 explains 62% of the variance and PC2 explains 17% of the variance. Abbreviations as in Figure S1.


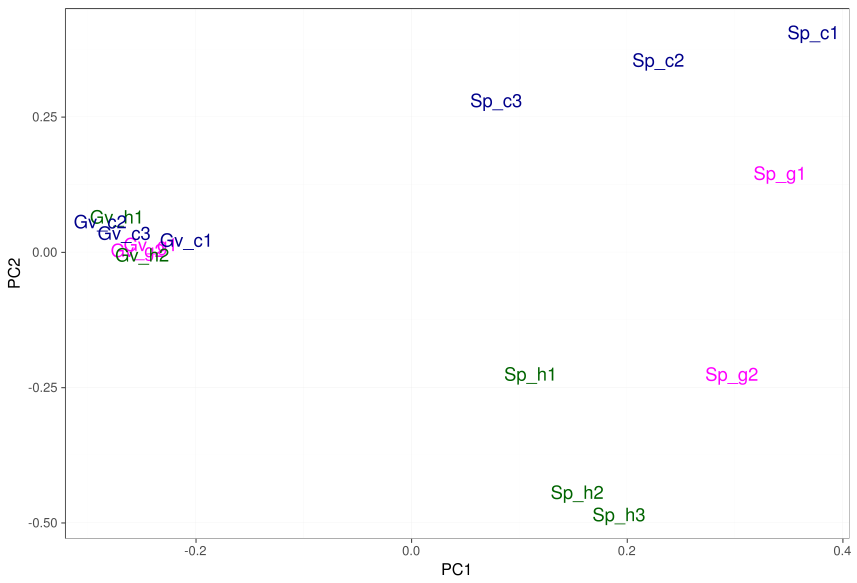


**Figure S3.** Principal component analysis (PCA) based on the Hellinger distance and relative OTU abundance. PC1 explains 22% of the variance and PC2 explains 15% of the variance. Abbreviations as in Figure S1.


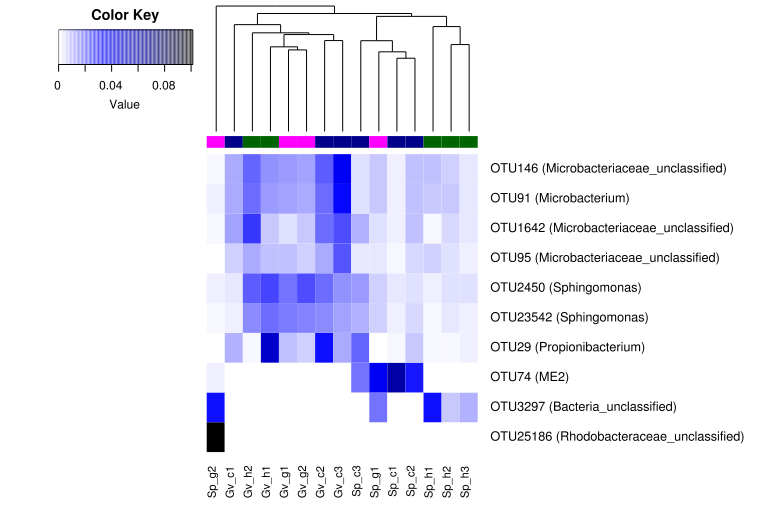


**Figure S4.** Hierarchical clustering based on the relative OTU abundance. The clustering was based on the Hellinger distances between samples. Only highly abundant OTUs are shown. Abbreviations as in Figure S1.


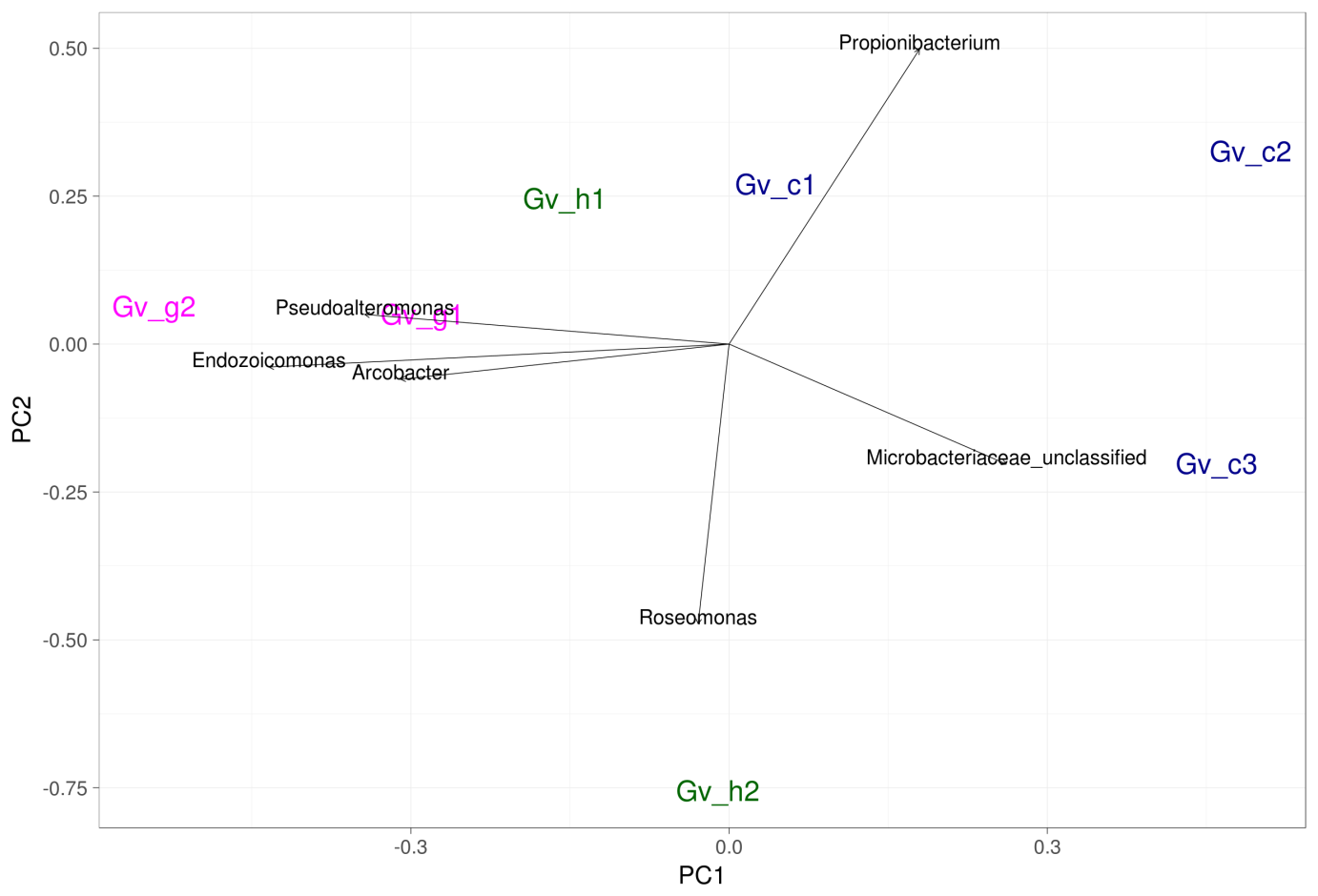


**Figure S5.** Principal component analysis (PCA) based on the Hellinger distance for samples from the Caribbean Sea. Abbreviations as in Figure S1. For PCA we merge OTU based on their genus-level taxonomy, if genus was not identified for OTU, then the lowest of identified taxonomic category was used. PC1 explains 30% of variance and PC2 explains 21% of variance. Taxa with the largest impact on PC1 and PC2 are shown as arrows.


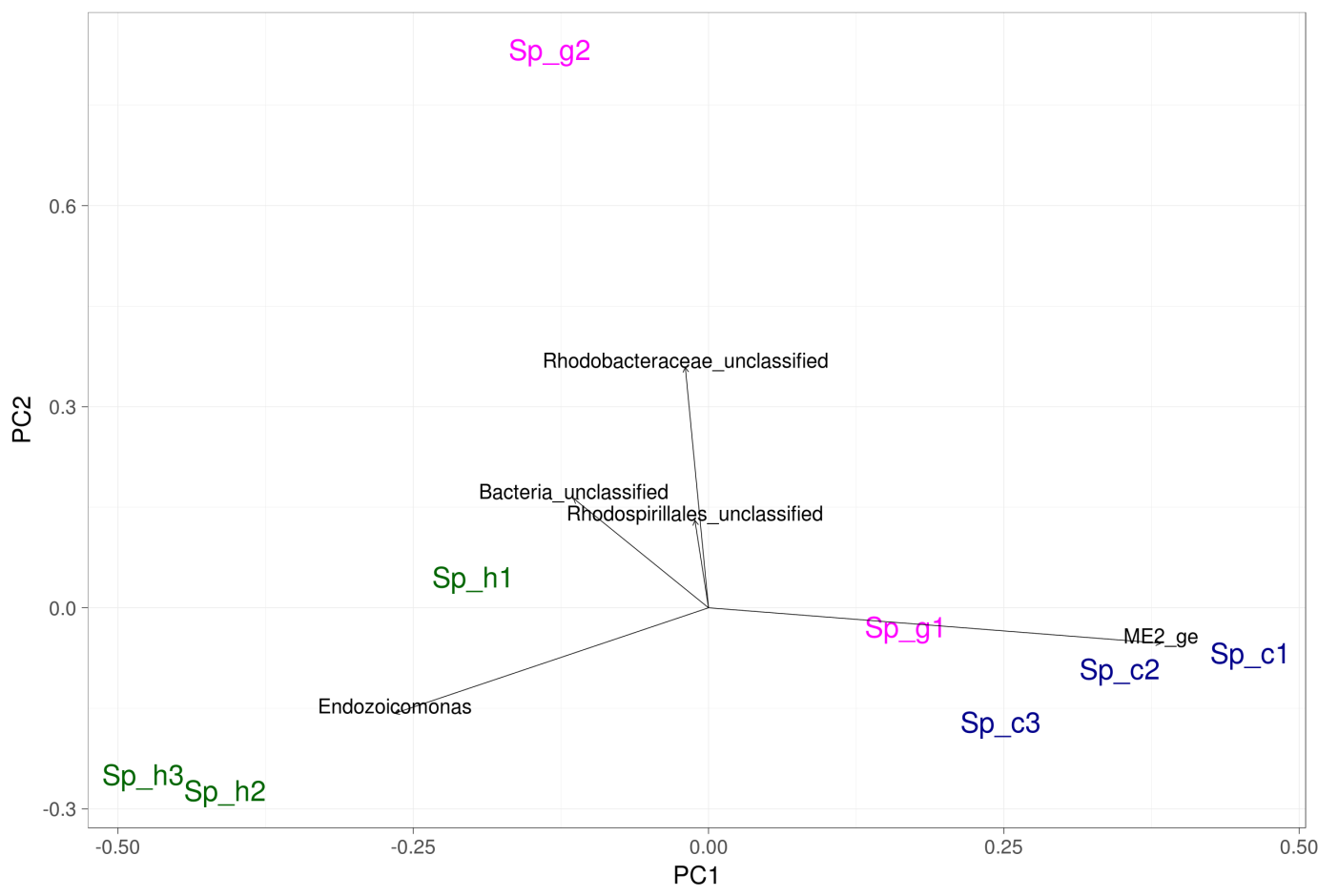


**Figure S6.** Principal component analysis (PCA) based on the Hellinger distance for samples from the Red Sea. Abbreviations as in Figure S4 and procedure as in Figure S5. PC1 explains 32% of the variance and PC2 explains 20% of the variance.
